# Investigation of the interaction between the MIR-503 and CD40 genes in irradiated U937 cells

**DOI:** 10.1186/1748-717X-7-38

**Published:** 2012-03-20

**Authors:** Guanghui Cheng, Shilong Sun, Zhanfeng Wang, Shunzi Jin

**Affiliations:** 1 Department of Radiation Oncology, China-Japan Union Hospital of Jilin University, Changchun 130033, China; 2 Ministry of Health Key Laboratory of Radiobiology, Jilin University, Changchun 130021, China; 3 Department of Neurosurgery, China-Japan Union Hospital of Jilin University, Changchun 130033, China; 4 Department of Neurosurgery, Beihua University, Jinlin 132011, China

**Keywords:** Radiation, miR-503, CD40

## Abstract

**Background:**

MicroRNAs (miRNAs) are a group of small noncoding RNAs that take part in diverse biological processes by suppressing target gene expression. Relatively few miRNAs have been studied in detail, especially miR-503, and hence the biological relevance of majority remains to be uncovered. Whether altered expression of miRNA-503 affects the immunity response to radiotherapy has yet to be addressed.

**Results:**

In the present study, we applied ionizing radiation with a dose of either 0.1 Gy or 5 Gy to irradiate U937 cells to confirm CD40 as a miR-503 target, which was identified using a bioimformatics tool. In high dose (5 Gy) ionizing-irradiated U937 cells, expression of miR-503 was up regulated while the expression of CD40 gene was down regulated. Using the transfection of the miR-503 gene into U937 cells and Luciferase assay, we confirmed that miR-503 suppressed the expression of CD40, and was a negtive regulator of CD40.

**Conclusions:**

To our knowledge, we are the first to describe involvement of miR-503 in radiobiological effect at a molecular level. This initial finding suggested the evidence that ionizing radiation could alter the expression of miR-503 and its target gene CD40, and may be very important to shed light on a possible mechanism regarding regulation of immune responses to irradiation.

## Background

Radiotherapy is a common adjuvant therapy for the treatment of patients with cancer. However, the effect of radiation on the immune system always leads to some side-effects. When living cells are exposed to ionizing radiation (IR), a series of alterations will take place, including transformation, cell cycle distress, mutations and chromosomal aberrations, abnormality of DNA repair and apoptosis [1,2]. The final outcomes of IR-exposing cells are determined by the activation of nuclear pattern [3]. Among the IR-responsive genes, those for the CD40 pathway have been brought significant attention to the field of radiobiological effect because of their prominent role in orchestrating both the humoral immune response and the cellular immune response [4]. The interaction of CD40 on the surface of B lymphocytes with its ligand (CD45), which is predominantly expressed by activated T cells, is critical for the induction of adaptive immunity by promoting the proliferation and differentiation of B lymphocytes into immunoglobulin-producing plasma cells. The CD40-CD154 interactions are also important in the activation of macrophages and the amplification of the innate immune response to intracellular and extracellular pathogens. Disruption of the CD40 pathway would therefore be predicted to confer deleterious effects on immune function. Indeed, mutations in the human CD154 gene results in the X-linked hyper IgM syndrome, a severe form of immune deficiency disorder that is clinically manifested by recurrent viral and bacterial infections and early lethality [5]. However, expression of the CD40 gene is not restricted to immune cells but also extends to a variety of other normal cell types, including fibroblasts, neuronal cells, epithelial and endothelial cells [6-8]; this widespread expression indicates that CD40 may play a crucial role in some physiological events and the pathogenesis of disease in humans.

Accumulative interest in MicroRNAs (miRNAs) over the past decade has uncovered their importance in several biological processes and has identified disease states with altered miRNA expression. miRNAs, a non-coding RNA family, are 19-25 nt transcripts that play a regulatory role in mRNA translation and degradation. There is a 2-8 nucleotide seed region within each miRNA, which is thought to be critical for target selection [9]. With this seed region, mature miRNAs selectively bind to mRNA recognition elements (MREs) within the 3'-UTR of target mRNAs. Different target genes may have several MREs and therefore be regulated by numerous miRNAs. Relatively few miRNAs have been studied in detail, especially miR-503, and hence the biological significance of majority remains to be uncovered. Microarray analysis is becoming a powerful tool for analysis of gene expression profiles. The comprehensive analysis of microRNA expression patterns in human dermal microvascular endothelial cells (HDMEC) irradiated by 2 Gy x-rays has recently been reported [10] and showed that the down-expression of miR-503 proberly takes part in the innate response mechanism of the endothelium to radiation. Zhou et al. [11] found that miR-503 can regulate metastatic function in hepatocellular carcinoma. Boominathan et al. [12] reported that tumor suppressors p53, p73 and p63 are very likely to regulate the processing of miRNAs such as let-7, miR-200c, miR-143, miR-107, miR-16, miR-145, miR-134, miR-449a, miR-503, and miR-21, although the precise mechanism of this action is largely unknown.

A number of studies have analyzed the transcriptional regulation of mRNAs and miRNAs in irradiated cells for better understanding of cellular responses to IR [13,14]. This study was undertaken to apply the Target scan and miRBase Registry program to investigate if function of the CD40 gene can be affected by radiation-induced miR-503 change in U937 cells that are malignant cells derived from human monocytes. The reason for selection of U937 cells as the primary test model was because monocytes could be involved in integrating innate and adaptive immune responses.

## Results

### Upregulation of miR-503 expression in irradiated U937 cells

**Figure 1:**
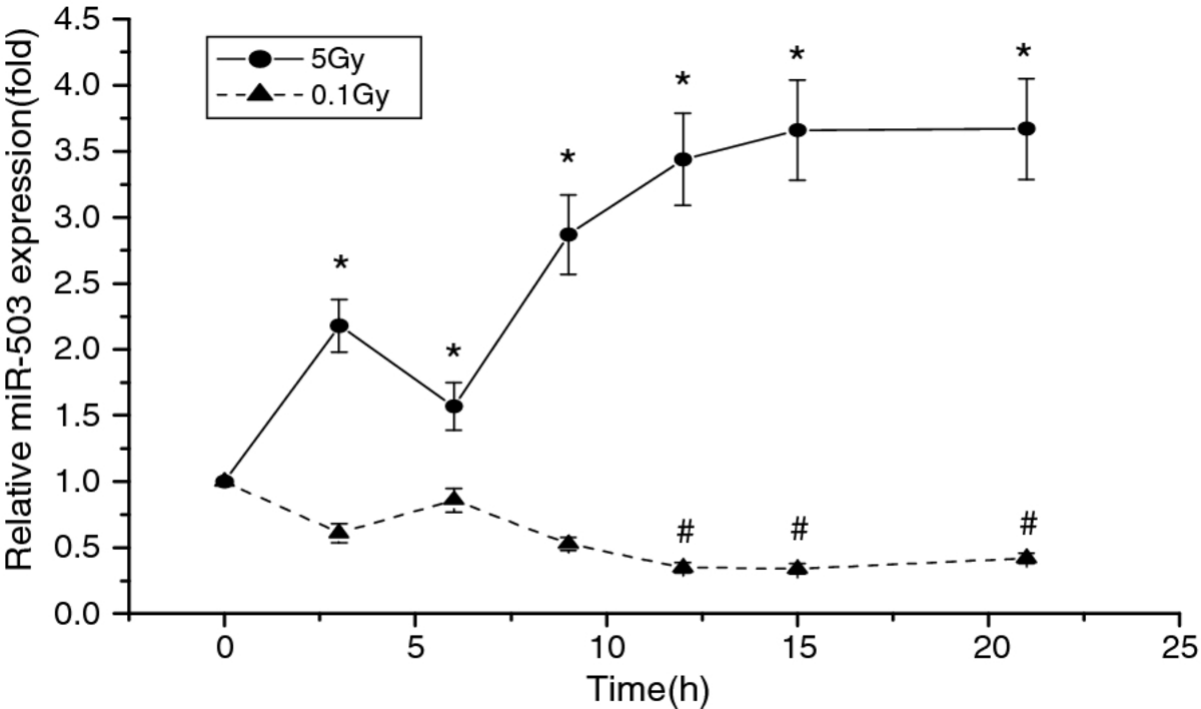
**Ionizing radiation stimulated miR-503 expression in the U937 cell line at different time points**. Quantitative PCR showed that following irradiation up regulation of miR-503 expression was observed in 5.0-Gy irradiated cells at 3 hours and afterwars, while downregulation of miR-503 regulation in 0.1-Gy irradiated cells at 12 hours and afterwards (*P < 0.01, # *P *< 0.01)

Following irradiation with either 0.1 Gy or 5 Gy, increased expression of the miR-503 gene was observed in the 5-Gy irradiated cells at 3 hours and afterwards, while reduced expression was observed in 0.1-Gy irradiated cells at 12 hours and afterwards (Figure 1). The altered miR-503 expression in the irradiated U937 cells suggested that miR-503 might play an important role in the response of living cells to radiation.

### Underexpression of the CD40 gene in irradiated U937 cells

**Figure 2:**
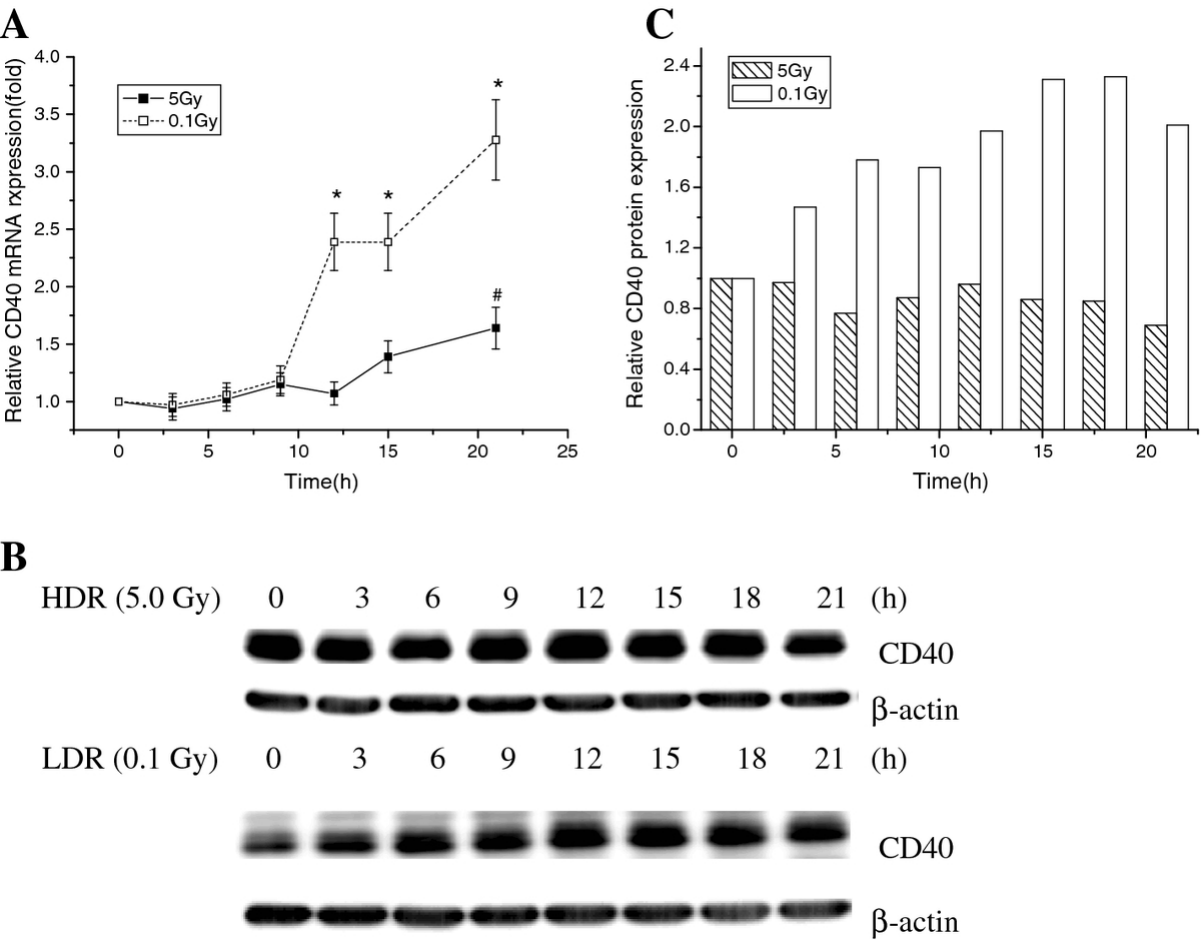
**Expression levels of mRNA and protein of CD40 in irradiated U937 cells**. (A) illustrates quantitative RT-PCR analysis of CD40 mRMA expression in irradiated U937 cells at various time points after IR exposure (*P < 0.01); (B) illustrates the levels of CD40 protein that was examined by western blot in in irradiated U937 cells at various time points after IR exposure; (C) illustrates CD40 protein expression in irradiated U937 cells at various time points after IR exposure(*P < 0.01).

To identify a possible target gene of miR-503, we computationally predicted targets using the miRBase Registry program [15] and the TargetScan program [16]. This analysis revealed that the CD40 gene might be a target of miR-503. Analysis of gene expression at different time points after radiation showed an inverse correlation in gene expression between CD40 and miR-503; the CD40 transcript was significantly overexpressed in 0.1-Gy irradiated cells as compared with that in 5-Gy irradiated cells (Figure 2A). Western blot analysis showed that CD40 expression also significantly increased in 0.1-Gy irradiated cells as compared with that in 5-Gy irradiated cells (Figure 1B). These results suggest that miR-503 may reduce CD40 expression at both the transcriptional and translational levels.

### CD40 is a target of miR-503

**Figure 3:**
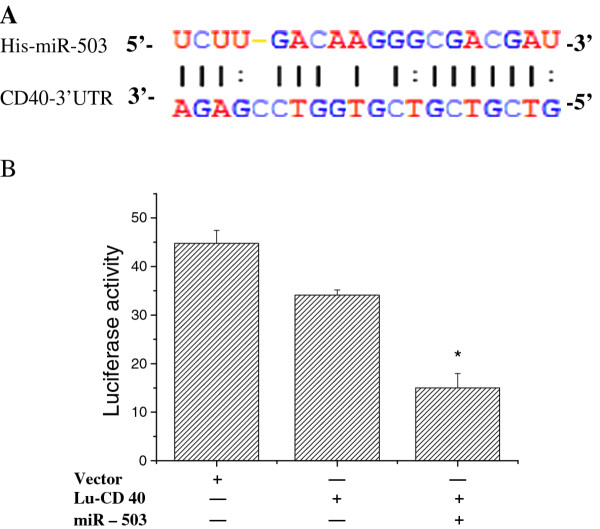
**Suppression of CD40 expression by miR-503**. (A) illustrates the miR-503 target sequence in the CD40 3'UTR that was predicted by Target Scan; (B) illustrates functional change of the cells transfected with the empty renilla luciferase reporter gene (psiCHECK2) or the reporter gene fused to the CD40 3' UTR. "+" represents transfection with a target gene and "-" no transfection. Results are expressed as relative light units (RLU) and were normalized with the luciferase activity expressed constitutively by the psiCHECK2 vector.

**Figure 4:**
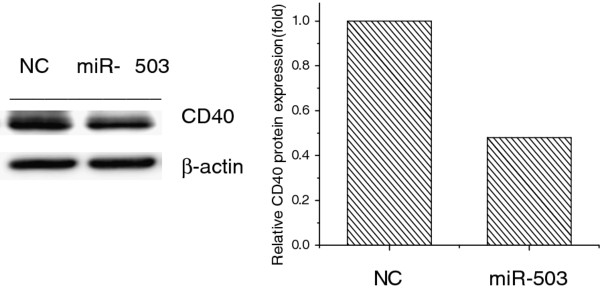
**Inhibition of CD40 protein expression in U937 cells transfected with the miR-503 gene**. (A) was the result from Western blot and (B) was the background.

Analysis with the miRBase Registry and TargetScan program showed that miR-503 might interact with CD40. As shown in Figure 3A, bases 29-35 of CD40 3'-UTR may be the miR-503 target site. Further analysis of the CD40 3'-UTR binding site for miR-503 with cloned 3'-UTR downstream of the renilla luciferase reporter gene confirmed that miR-503 was likely to target the CD40 gene due to the decrement in the renilla activity (Figure 3B). As shown in Figure 4, transfection of the miR-503 gene into U937 cells induced inhibition of CD40 protein expression by 45%.

## Discussion

In this study, we applied two doses of radiation (0.59 Gy/min for 5 Gy radiation and 67 cGy/min for 0.1 Gy radiation) to irradiate U937 cells. The CD40 gene was identified as a target of miR-503 using bioinformatic tools and further confirmed by experimental validation. Because a target locus predicted by combination of algorithms may have more functional relevance than that predicted using a single algorithm alone, we analyzed a range of databases. CD40 was listed as a predicted target in both of the target prediction databases interrogated. We have experimentally validated CD40 as a target of miR-503 by showing that overexpression of miR-503 resulted in a decrease of CD40 mRNA expression, protein production and luciferase activity in a reporter system that contains the full length CD40 3'-UTR, suggesting direct targeting by miR-503. We also presented a functional role of CD40 in the signaling pathways induced by radiation and combined this observation with regulation of CD40 by miR-503 to draw a conclusion.

The present results suggest that miR-503 may interact with CD40 and reduce CD40 expression at both the transcriptional and translational levels. If this were a case, miR-503 could play an important role in the response to ionizing radiation through the inhibition of CD40. Analysis of expression profiling has revealed a pattern of altered miRNA expression in a variety of human diseases. Abnormal expression of a number of miRNAs has been observed in a variety of tumors (e.g., miR-34a, miR-143, miR-145, miR-21) [17,18]. Several miRNAs are also specifically expressed in some types of cancers [19,20]. Increasing evidence for important roles of miRNAs in regulating immunity system has been reported [21-23]. The present work gave further evidence that radiation-induced miRNA expression profiles may represent a specific response that can trigger a cascade effect on the immune system. However, this hypothesis needs further investigation.

To our knowledge, we are the first to report the involvement of miR-503 in radiation-induced biological response. In particular, we observed the altered expression of miR-503 that is very likely to act as a negative regulator of the CD40 gene. Our findings have important implications regarding regulation of immune responses to ionizing radiation, which may be useful to improve clinical effectiveness of radiotherapy for the treatment of malignant tumors.

## Conclusions

In high dose (5 Gy) ionizing-irradiated U937 human monocyte cells, expression of miR-503 was up regulated while the expression of CD40 gene was down regulated. Analysis with the miRBase Registry and TargetScan program showed that miR-503 might interact with CD40. Using the transfection of the miR-503 gene into U937 cells and Luciferase assay, we confirmed that miR-503 suppressed the expression of CD40, and was a negtive regulator of CD40. Together, This initial finding suggested the evidence that ionizing radiation could alter the expression of miR-503 and its target gene CD40.

## Methods

### Cell culture and irradiation

U937 cells were maintained in RPMI 1640 medium and supplemented with 10% fetal bovine serum, 100 U/ml penicillin, 100 μg/ml streptomycin, and 2 mM L-glutamine. The cultured cells were divided into two groups, the high dose irradiated group and the low dose irradiated groups. The irradiated groups was exposed to radiation of either 0.1 Gy or 5 Gy using a 4-MV linear accelerator (Clinac 4/100, Varian, Palo Alto, CA).

### Quantitative RT-PCR analysis

Total RNA was extracted from the cell lines using the TRIzol method according to the manufacturer's protocol [24]. Total RNA was reverse transcribed to cDNA using Superscript II reverse transcriptase (Invitrogen) and oligonucleotide primers. Quantitative real-time PCR (RT_PCR) analysis of gene expression was performed in a 25-ul reaction volume containing cDNA, SYBR Premix Ex Taq (Takara Bio Inc., Shiga, Japan), TaqMan Universal PCR Master Mixture and primers for each gene. Quantitation of miRNAs was carried out using TaqMan microRNA assays (Applied Biosystems, Foster City, CA). The PCR amplification was conducted in reaction mixture using the TaqMan Universal PCR Master Mixture according to the protocol supplied by the manufacturer. Samples were analyzed with the ABI PRISM 7000 sequence detection system (Applied BioSystems); the specificity of PCR reaction was determined by melting curve analysis at the dissociation stage. Specific primers for the CD40 gene were as follows: 5'-TCTGCACCTGTGAAGAAGGC-3' (forward) and 5'-CACATTGGAGAAGAAGCCGA-3' (reverse); the GAPDH was used as a reference gene and its PCR primers included 5'-GAAGGTGAAGGTCGGAGTC-3' (forward) and 5'- GAAGATGGTGATGGGATTTC-3' (reverse). The relative quantitative method was used for the quantitative analysis and fold change (FC) was used to present data.

### Western blotting

U937 cells were harvested and lysed at a designed time point of post-radiation; proteins were separated on a SDS/polyacrylamide gel and transferred into a PVDF membrane (Bio-Rad, Hercules, CA). After blocking, the membranes were incubated with the primary antibody, anti-CD40 polyclonal antibody (Santa Cruz Biotechnology, Santa Cruz, CA). The membranes were extensively washed and incubated with a horseradish peroxidase-conjugated secondary antibody (Bio-Rad). The antigen-antibody complexes were visualized by West-Q-Chemiluminescent Sub Kit Plus (BIOTANG, Waltham, MA).

### Constructs and transfection

The precursor of the hsa-miR-503 was amplified from U937 genomic DNA using the primers 5'-CTCGTGGGGAAGGTAGAAGG-3' and 5'-GGGAAAGGGACGAGTCCATC -3', and the resulting products were cloned into EcoR1/Not1 restricted pcDNA DEST47 (Invitrogen, Carlsbad, CA). The expression vector for CD40 was kindly provided by Jeong et al. (2009), and the construct was transfected using FuGENE HD (Roche Applied Science, Mannheim, Germany) according to the manufacturer's protocols.

### Luciferase assay

The 3'-UTR of the CD40 gene was fused to the renilla gene using the XhoI/NotI restriction sites of the psiCHECK2 vector 4 (Promega). A total of 8 × 10 U937 cells were co-transfected with 30 ng of the indicated vector and 90 ng of the pcDNA DEST47 cloned miR-503 using Fugene (HD, Roche) for 48 h. Luciferase assays were performed using the Dual-Luciferase assay (Promega). Normalization of the Renilla expression was performed using the luciferase gene present on the psiCHECK2 vector.

### Statistical analysis

All the statistical tests were performed using the SPSS for Windows 11.0 software package (SPSS, Chicago, IL, USA), including descriptive statistics, one-way analysis of variance (ANOVA) and two-way ANOVA. The significant level was set at a P-value of < 0.05.

## Abbrevations

IR: Ronizing radiation; ANOVA: One-way analysis of variance.

## Competing interests

The authors declare that they have no competing interests.

## Authors' contributions

GC performed the qRT-PCR, Luciferase assay, cultured cell lines, and performed some of the bioinformatics analyses. ZWperformed the qRT-PCR, some bioinformatics analyses, all statistics, and helped revise the manuscript. SS helped draft and revise the manuscript, and assisted with supervision of experiments. SJ conceived the idea, drafted the manuscript, supervised the experiments, and performed some of the imaging. All authors read and approved the final manuscript.

## Supplementary Material

Additional file 1 **Figure S1**. Expression levels of CD40 protein in irradiated DC cells, Figure S2. Suppression of CD40 expression by different miRNA. Figure S3. Heatmap illustrating expression of miRNAs in response to irradiation in H1299 cells. Figure S4. Relative luciferase activity in the U937 Cells transfected with the empty renilla luciferase reporter gene (psiCHECK2) and the U937 Cells co-transfected with psiCHECK2 and miR-503. Table S1. Up-regulated miRNAs in different radiation sensitivity cell lines. Click here for file
