# Supplementary material for: Investigation of the interaction between the MIR-503 and CD40 genes in irradiated U937 cells
Source: Radiat Oncol. 2012 Mar 20;7:38. doi: 10.1186/1748-717X-7-38 (PMC3325872; doi:10.1186/1748-717X-7-38)
Supplement: Additional file 1 — Figure S1. Expression levels of CD40 protein in irradiated DC cells, Figure S2. Suppression of CD40 expression by different miRNA. Figure S3. Heatmap illustrating expression of miRNAs in response to irradiation in H1299 cells. Figure S4. Relative luciferase activity in the U937 Cells transfected with the empty renilla luciferase reporter gene (psiCHECK2) and the U937 Cells co-transfected with psiCHECK2 and miR-503. Table S1. Up-regulated miRNAs in different radiation sensitivity cell lines. [file 1748-717X-7-38-S1.DOC]

Supplementary Figure 1. Expression levels of CD40 protein in irradiated DC cells.

**Lu-CD40 + + + +**

**miR 503 — + — —**

**miR-203 — — + —**

**miR-29b — — + —**

Supplementary Figure 2. Suppression of CD40 expression by different miRNA.

**
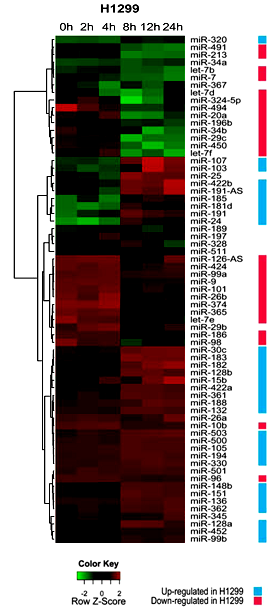
**

Supplementary Figure 3. Heatmap illustrating expression of miRNAs in response to irradiation in H1299 cells

Supplementary Figure 4. Relative luciferase activity in the U937 Cells transfected with the empty renilla luciferase reporter gene (psiCHECK2) and the U937 Cells co-transfected with psiCHECK2 and miR-503.

| Supplementary Table 1. Up-regulated miRNAs in different radiation sensitivity cell lines  **Up-regulated microRNAs** | |  |
| --- | --- | --- |
| **H460** | **H1299** | |
| hsa_miR_320, hsa_miR_197, hsa_miR_328, hsa_miR_96, hsa_miR_331, hsa_miR_7, hsa_miR_98 (7) | hsa_miR_128b, hsa_miR_362, hsa_miR_132, hsa_miR_30c, **hsa_miR_503**, hsa_miR_185, hsa_miR_183, hsa_miR_422a, hsa_miR_361, hsa_miR_136, hsa_miR_148b, hsa_miR_128a, hsa_miR_105, hsa_miR_151, hsa_miR_107, hsa_miR_194, hsa_miR_191, hsa_miR_423, hsa_miR_320, hsa_miR_181d, hsa_miR_224, hsa_miR_330, hsa_miR_24, hsa_miR_524, hsa_miR_302b (25) | |
